# Reverse Posterior Interosseous Artery Flap: Indications, Technique, and Complications in 18 Consecutive Cases

**DOI:** 10.1055/s-0045-1812467

**Published:** 2025-12-10

**Authors:** Alvaro Baik Cho, Antonio Isidoro de Sousa Neto, Kríssia Caroline Soares Franco, Marcelo Rosa de Rezende, Teng Hsiang Wei, Rames Mattar Junior

**Affiliations:** 1 Hand Surgery and Reconstructive Microsurgery Group, Instituto de Ortopedia e Traumatologia, Hospital das Clínicas da Faculdade de Medicina da Universidade de São Paulo, São Paulo, SP, Brazil; 2 Orthopedics and Traumatology Department, Faculdade de Medicina da Universidade de São Paulo, São Paulo, SP, Brazil

**Keywords:** fascia/transplantation, hand injuries, soft tissue injuries, surgical flaps, fáscia/transplante, lesões dos tecidos moles, retalhos cirúrgicos, traumatismos da mão

## Abstract

**Objective:**

To evaluate the success rate of the reverse posterior interosseous artery flap, inferring its predictability, reliability, and safety.

**Methods:**

We conducted a retrospective observational study including 18 patients with soft-tissue lesions in the distal third of the upper limb who underwent posterior interosseous artery flap surgery. The study analyzed the flap size, the recipient area, the need for additional grafting, the clinical outcome, and the complications.

**Results:**

There was one total and one partial loss of the flap, resulting in a success rate of 94.45%. Most cases healed well, and we did not observe major complications. More than half (55.5%) of the cases required grafting. In four cases, the flap was insufficient to cover the defect, requiring skin grafting in areas of granulation. In four cases, we performed thumb coverage, which is essential for hand function. The average flap size was of 9.6 cm in length by 4.4 cm in width, with the length ranging from 5.5 to 13 cm, and the width, from 3 to 7 cm. The flap was beneficial in functional areas such as the thumb and first commissure.

**Conclusion:**

The posterior interosseous artery flap is a safe and effective option to cover defects in the distal third of the upper limb, especially on the dorsum of the hand and thumb, with low morbidity and good functional and esthetic outcomes.

**Level of Evidence:**

IV, case series.

## Resumo

**Objetivo:**

Avaliar a taxa de sucesso do retalho reverso da artéria interóssea posterior e inferir sua previsibilidade, confiabilidade e segurança.

**Métodos:**

Conduzimos um estudo retrospectivo observacional de 18 pacientes com lesões de partes moles no terço distal do membro superior submetidos a retalho da artéria interóssea posterior. Foram analisados o tamanho do retalho, a área receptora, a necessidade de enxertia complementar, o resultado clínico e as complicações.

**Resultados:**

Houve uma perda total e uma parcial, o que resultou em taxa de sucesso de 94,45%. A maioria dos casos apresentou boa cicatrização e ausência de complicações importantes. Foi necessária enxertia em 55,5% dos casos. Em quatro casos, o retalho mostrou-se insuficiente para a cobertura da falha, sendo necessário enxerto cutâneo em áreas de granulação. Em quatro casos, foi realizada cobertura de polegar, que é fundamental para a funcionalidade da mão. A média do tamanho foi de 9,6 cm de comprimento por 4,4 cm de largura, com variação de comprimento de 5,5 a 13 cm e de largura, de 3 a 7 cm. O retalho demonstrou utilidade em áreas funcionais como o polegar e a primeira comissura.

**Conclusão:**

O retalho da artéria interóssea posterior é uma opção segura e eficaz para a cobertura de falhas no terço distal do membro superior, especialmente no dorso da mão e no polegar, com baixa morbidade e bons resultados funcionais e estéticos.

**Nível de Evidência:**

IV, série de casos.

## Introduction

Soft tissue defects in the hand are challenging injuries, as they often present with exposure of tendons or neurovascular structures, 1 and they require proper skin coverage for early rehabilitation to prevent stiffness and contractures.

The literature describes several coverage options, including local, pedicled, or microsurgical flaps. The surgeon's preference and comfort are key elements in flap selection, considering factors such as the degree of technical difficulty, pedicle length, volume, and potential for primary closure.

The single-stage reconstruction of these defects results in early mobilization, reduces hospital stays, minimizes infection rates, and yields good functional outcomes. Regional flaps offer these advantages, reducing the surgical time and presenting fewer technical difficulties compared with microsurgical flaps. 2

The most common regional flaps for these cases are the antebrachial fasciocutaneous flaps with a retrograde flow from the radial artery, the ulnar artery, and the posterior interosseous artery (PIA). The first two present the disadvantage of sacrificing significant hand arteries, increasing donor site morbidity. Another disadvantage and limitation of these flaps is the need to preserve the palmar arch to ensure its perfusion. Palmar arch compromise is not uncommon in severe hand injuries.

Two independent groups first described the PIA flap for coverage of elbow defects. 3 4 5 The PIA flap started to be used in distal defects after its description for retrograde flow, 6 7 based on its anastomosis with the anterior interosseous artery, presenting itself as an interesting option for coverage due to its reliability and predictability, 8 especially in the dorsal region of the hand up to the proximal interphalangeal joints and the first commissure area. 9

The present study aimed to evaluate the success rate of the PIA flap, inferring its predictability, reliability, and safety.

## Methods

The institutional Ethics Committee approved the study under opinion number 7.362.990/2025 (CAAE: 85725225.2.0000.0068).

The current is a primary, retrospective, observational, and descriptive study of a series of 18 consecutive cases of patients with loss of skin coverage in the distal third of the wrist, hand, and first commissure, secondary to trauma or infection. The patients underwent surgery from September 2001 to June 2023, and the same surgeon, a specialist in hand and microsurgery, performed all procedures.

Flap indication resulted from the need for coverage after amputation or coverage failure. Amputation occurred at the carpal level in three cases, at the first metacarpal level in one case, and at the level of the interphalangeal joint of the thumb in one case. The coverage failure site was the dorsal region of the hand in five patients, the dorsal region of the hand at the first metacarpal level in three cases, the dorsoradial region of the wrist in one subject, the volar radial region of the wrist in one case, the first web space in one patient, and the thenar region in one case. One patient required coverage due to scar retraction at the first web space.

We evaluated data such as the size of the defect and the flap, the recipient sites and lesion severity, the need for skin graft at the donor and recipient areas, and the complications (infection and total or partial flap loss). We excluded patients who underwent other coverage options, such as regional or microsurgical flaps.

## Surgical Technique

First, the surgeon performed the anatomical markings, drawing a line from the lateral epicondyle of the distal humerus to the distal radioulnar joint (DRUJ). Next, the surgeon marked a point 2 cm close to the DRUJ, indicating the probable location of the anastomosis between the PIA and the anterior interosseous artery, as the rotation point of the flap pedicle.

**Fig. 1:**
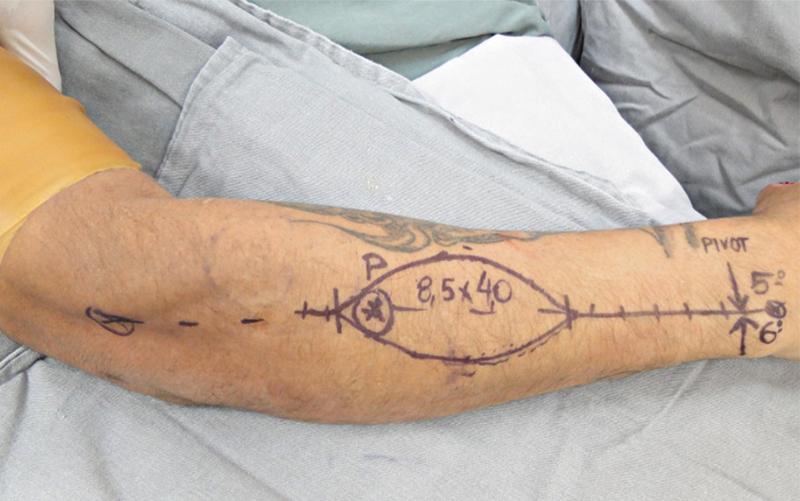
Anatomical markings.

**Fig. 2:**
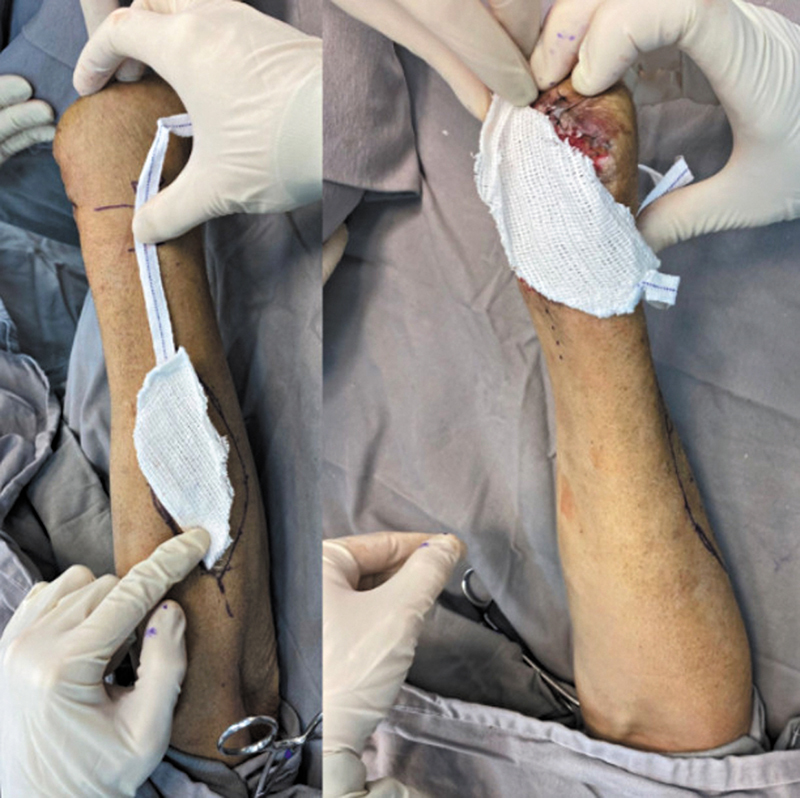
Template for flap rotation.

Then, the surgeon divided the original line into three equal parts; the middle third portion represented the ideal location for fasciocutaneous flap elevation, as it is the site of the most relevant perforator ( Fig. 1 ). The surgeon debrided the coverage defect and measured the distance between the rotation point of the flap and the defect: this measurement determined the length of the flap and pedicle for dissection ( Fig. 2 ).

**Fig. 3:**
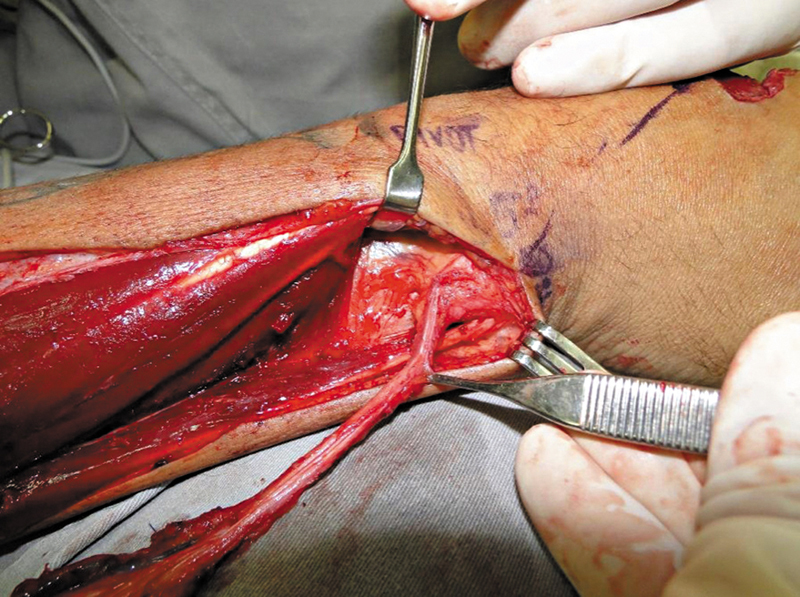
Pedicle dissected up to the point of rotation.

**Fig. 4:**
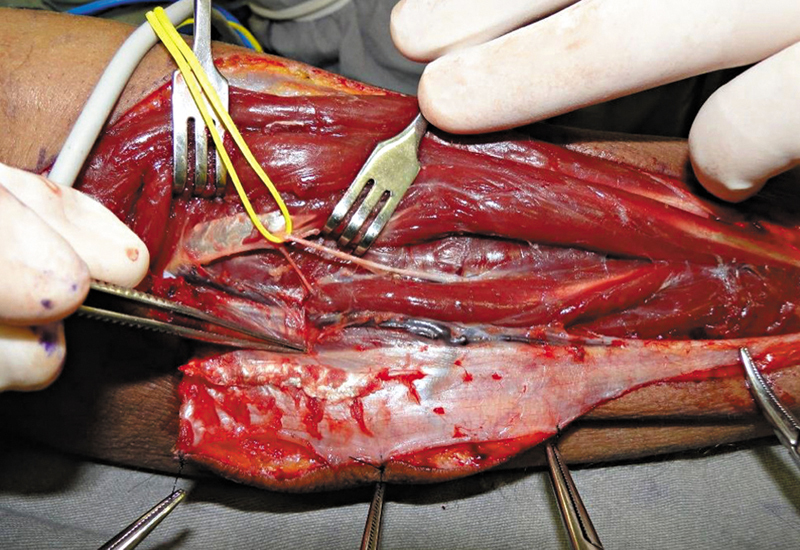
Posterior interosseous nerve separated from the pedicle.

Dissection always began with a distal incision and the identification of the tendons of the extensor carpi ulnaris (sixth extensor compartment) and the extensor digiti minimi (fifth compartment). The PIA pedicle was between these tendons. Next, the surgeon made a longitudinal incision on the fascia over these tendons and carefully retracted them to identify and confirm the presence of the artery. In all cases, the artery was closest to the fifth compartment tendon. Next, dissection proceeded from distal to proximal up to the point of rotation ( Fig. 3 ). The posterior interosseous nerve was carefully dissected from the artery ( Fig. 4 ). No case required the section of any branch of the nerve.

**Fig. 5:**
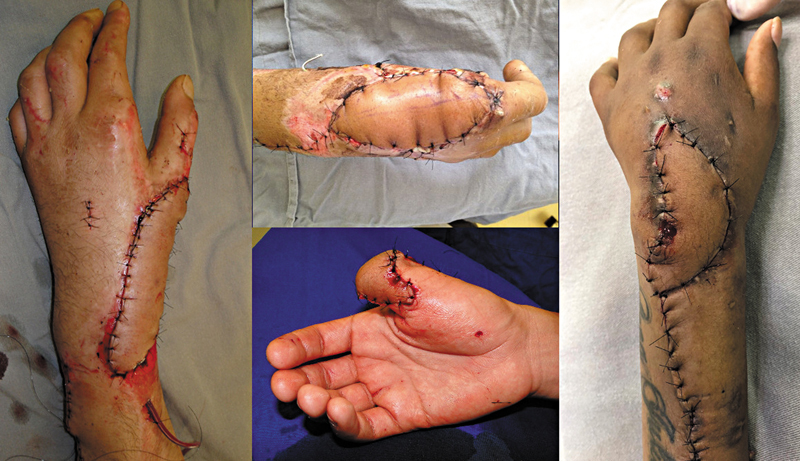
Soft tissues defects covered with the flap.

The flap was sufficient to cover exposed noble structures in all patients ( Fig. 5 ). However, some cases required a partial skin graft in flap-associated marginal areas for complete lesion coverage. Whenever possible, the surgeon performed the primary closure of the donor site. If necessary, the donor-site closure included a partial skin graft. In 12 cases, the flap passed through a subcutaneous tunnel, while 6 cases required an incision.

After the surgical procedure, the flap status was regularly checked, using color, bleeding, and capillary refill time as parameters. The patients underwent antebrachiopalmar immobilization for 2 weeks for flap autonomization. Next, they underwent a monthly functional monitoring for a variable follow-up period.

## Results

The present study comprised 18 surgical procedures. Of these, 17 coverage failures occurred due to traumatic events, and 1 failure may have resulted from pyoarthritis of the wrist ( Table 1 ). Most patients were male. Injuries were more common in the right hand (11 cases) compared with the left hand (7 cases).

**Table 1:** Descriptive aspects of cases receiving posterior interosseous artery flaps in the upper limbs

Cases ( *n* = 18)	Sex	Coverage failure site ^*^	Size (cm) ^***^	Donor area closure	Graft in the recipient area ^***^	Outcome	Etiology
1	Male	First metacarpal amputation	5.5 × 4.0	Primary	–	Favorable	Trauma
**2**	Male	Amputation at the carpal level	8.0 × 4.5	Grafting	–	Favorable	Trauma
**3**	Male	Dorsum of the hand	6.5 × 4.0	Primary	–	Favorable	Pyoarthritis
**4**	Male	Dorsum of the first metacarpal	9.0 × 5.0	Grafting	–	Favorable	Trauma
**5**	Male	Radial dorsum of the wrist and hand	12 × 4.5	Grafting	–	Favorable	Trauma
**6**	Male	Thenar region	9.0 × 4.0	Grafting	Yes	Favorable	Trauma
**7**	Male	Dorsum of the hand	12.0 × 7.0	Grafting	–	**Partial loss**	Trauma
**8**	Male	Thumb, at the level of the interphalangeal joint	9.0 × 4.0	Primary	–	Favorable	Trauma
**9**	Male	Amputation at the carpal level	13.0 × 4.5	Grafting	Yes	Favorable	Trauma
**10**	Male	Amputation at the carpal level	13.0 × 4.5	Grafting	Yes	Favorable	Trauma
**11**	Male	Radial dorsum of the hand	11.0 × 5.0	Grafting	–	Favorable	Trauma
**12**	Male	Dorsum of the first metacarpal	13.0 × 5.5	Primary	–	Favorable	Trauma
**13**	Male	Dorsum of the first metacarpal	8.5 × 4.0	Primary	–	Favorable	Trauma
**14**	Male	Dorsum of the hand	7.5 × 4.0	Grafting	–	**Total loss**	Trauma
**15**	Female	Radial dorsum of the wrist	7.0 × 3.0	Primary	–	Favorable	Trauma
**16**	Male	Volar radial region of the wrist	12.5 × 5.0	Primary	–	Favorable	Trauma
**17**	Male	First web space	5.5 × 4.0	Grafting	–	Favorable	Trauma
**18**	Female	First web space	11.0 × 3.0	Primary	–	Favorable	Trauma

Notes:^*^ Indication or location of coverage failure; ^**^ flap in centimeters (length x width); ^***^ supplementary grafting to the flap in the recipient area.

Regarding size, the largest flap measured 13 × 5.5 cm, and the smallest, 7 × 3 cm. The average flap size was of 9.6 × 4.4 cm. Thus, the flaps ranged from 5.5 cm to 13 cm in length and from 3 cm to 7 cm in width ( Table 1 ).

**Fig. 6:**
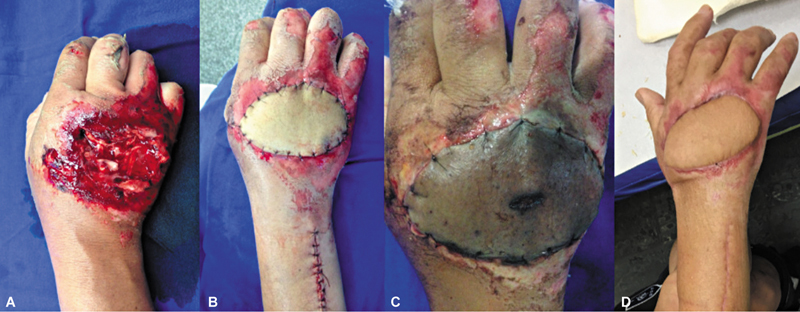
( **A** ) Initial soft tissue defect; ( **B** ) aspect immediately after covering with a posterior interosseous artery flap; ( **C** ) total flap necrosis; and ( **D** ) aspect in the late postoperative period of covering with a posterior interosseous artery flap; the patient presents with wrist flexion-extension and preserved fingers.

As for the outcomes, flap loss was total in 1 case (5.55%) and partial in another case (5.55%). The total loss occurred in a patient with coverage failure on the dorsum of the hand due to trauma. The removal of the necrotic area of the flap and the creation of a pedicled radial artery antebrachial flap led to a satisfactory outcome ( Fig. 6 ). The partial loss case also involved coverage failure on the dorsum of the hand due to trauma, with partial peripheral necrosis of the distal and radial edges of the flap after epidermolysis. Follow-up with healing by secondary intention was chosen. In terms of effectiveness in covering the exposed area, only the flap with total loss failed its function, resulting in a success rate of 94.45%.

**Fig. 7:**
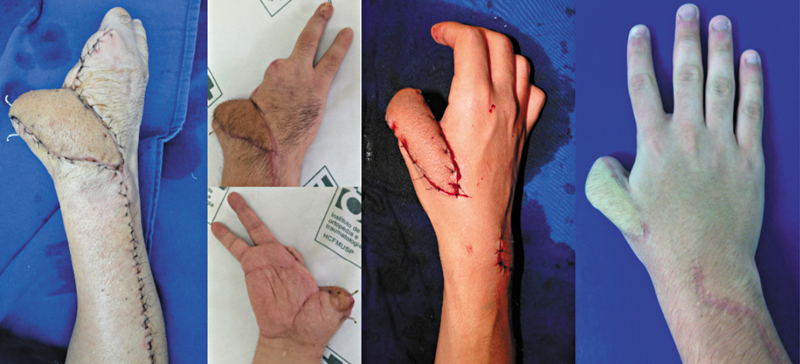
Thumb flaps.

We did not assess the hand or wrist function, since it depends on variables not related to the use of flaps, particularly the initial severity of the trauma. However, in four cases, the flaps were effective in maintaining limb function, especially in severe cases of partial thumb amputation, enabling the preservation of the maximum possible digit length and movements, such as pinch and opposition ( Fig. 7 ). In total, 6 (33.3%) patients received a flap for thumb region coverage.

All donor sites healed without complications. There were no reports of infection, dehiscence, nor compromise of the venous return or the posterior interosseous nerve. The PIA flap was insufficient to completely cover the defect in four cases, requiring a skin graft in areas with no exposed tendons or neurovascular structures. Regarding primary closure of the donor site, 10 (55.5%) cases needed grafting.

## Discussion

Cheema et al. 10 reported their experience with 64 flaps, 88.24% of which survived completely; the four flaps that failed in their study presented pedicle skeletonization. Büchler e Frey 11 reported 4 partial losses in 16 cases, all due to surgical site infection. Lee et al. 12 reported 2 losses in 49 cases, both secondary to venous congestion.

In the current study, 16 patients recovered well, while 1 had total and 1 presented with partial flap necrosis. In the case of total necrosis, the pedicle suffered an injury during dissection, and, in the case of partial flap loss, it is estimated that the flap design was excessively proximal due to the need for a long rotation arch.

Thus, it is possible to estimate that the flap is safe for first web space defects and, especially, for the dorsum of the hand. If flap rotation is necessary in regions distal to the metacarpophalangeal joint of the long fingers or beyond the first commissure, as in volar defects, it may not be the best option due to the risk of loss. However, Wu et al. 13 described eight cases of successful coverage of defects in long fingers.

**Fig. 8:**
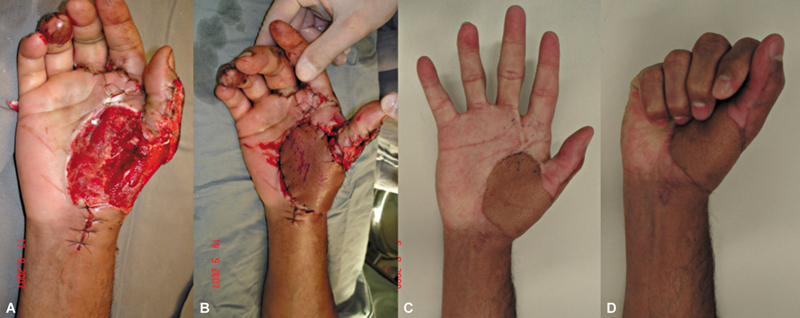
( **A** ) Initial soft tissue defect; ( **B** ) aspect immediately after covering; and ( **C** , **D** ) functionality 11 months after surgery.

The volar region of the hand with the best flap coverage capacity was the thenar region, with one case achieving good coverage of the recipient region and a good functional prognosis ( Fig. 8 ). We do not recommend palm areas beyond this limit for coverage.

**Fig. 9:**
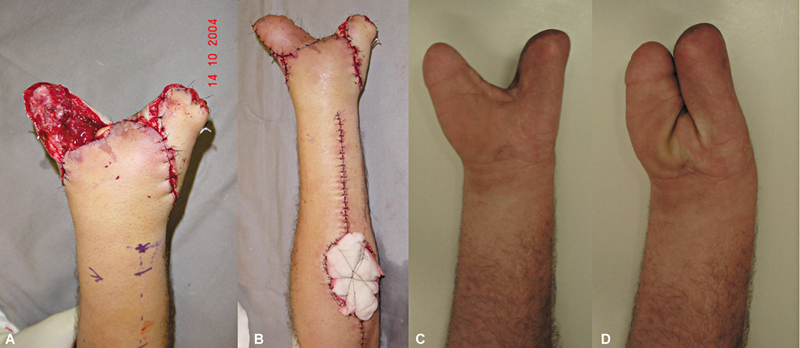
( **A** ) Initial soft tissue defect; ( **B** ) aspect immediately after covering; and ( **C,D** ) functionality 3 years after surgery.

The flap was also essential for the functional prognosis of the limb in cases of mutilating hand injuries. In one case with complex loss of hand structures, the flap was created to provide bony coverage to the radius, enabling the patient to perform a pinch movement ( Fig. 9 ).

**Fig. 10:**
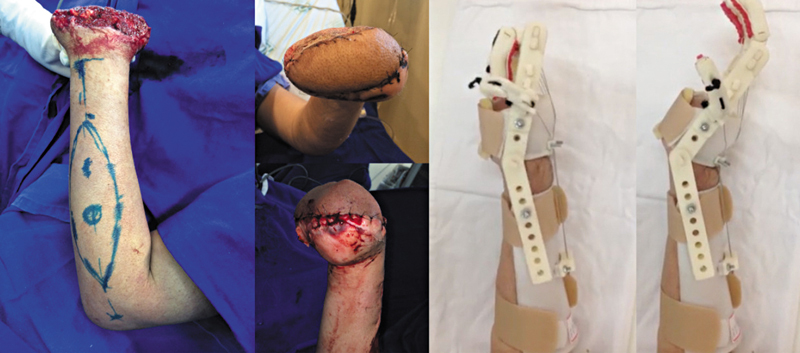
Flap to help the prosthetic fitting after a complex hand amputation.

In a severe case of bilateral traumatic amputation at the carpal level, we used the flap for more robust coverage of the bilateral carpus. This coverage was an initial stage for a more adequate prosthetic fitting in the future, a factor that directly influences the functionality of the affected limb ( Fig. 10 ).

The literature shows wide variations in flap sizes. Zhou et al. 14 used different flap sizes; the largest flap was of 11 × 8.5 cm, and the smallest, 5.3 × 2.6 cm. In the series by Kumaraswamy et al., 15 the largest flap measured 10 × 4 cm. Mago 16 described a series of 20 cases, with 2 cases of total loss, with sizes ranging from 4 × 4 cm to 10 × 8 cm. Balakrishnan et al. 17 used the smallest flap, measuring 5 × 2.5 cm, and the largest, measuring 21 × 10 cm. Ren et al. 8 considered flap width as the greatest limiting factor for its use, with a risk of loss in flaps larger than 6 cm; the authors achieved primary closure in 16.7% of cases in a sample of 30 patients. In the present study, one flap was wider than 6 cm (7 cm), and it did not suffer any loss. However, it is worth emphasizing that absolute measurements do not consider the dimensions of each patient's forearm, which vary according to the body type.

As reported by several authors, dissection should begin distally, and surgeons should seek the anastomosis of the perforating vessel between the PIA and the anterior interosseous artery at least 2 cm proximal to the dorsal wrist crease. The current study followed the same principle, and we found the perforating vessel in all cases.

Although surgeons should be aware of anatomical variations in vessels, few authors have reported their experiences with them. During dissection, Penteado et al. 4 found no anterior interosseous artery beyond the middle third of the forearm in four cases, and no anastomotic perforator in one case. In 2 out of 36 cases, Büchler e Frey 11 found no PIA beyond the middle third of the forearm. In the current study, we found a single anatomical variation (a duplicated pedicle), which did not negatively affect the dissection or outcome of the case.

The disadvantage of the PIA flap is the technical difficulty of dissection due to its thin and delicate pedicle. Furthermore, it requires identification and neurolysis of the posterior interosseous nerve to avoid finger-extension deficits. As an advantage, this flap presents good esthetic acceptance, as the thickness of the dorsal skin from the forearm is similar to that of the dorsal skin of the hand. All patients in the present study were satisfied with the cosmetic outcome.

The current study has limitations, including its small sample size and retrospective nature, which prevent drawing definitive conclusions, unlike studies with higher levels of evidence. However, it was possible to estimate parameters that aid in the surgical technique and indicate the best choice of recipient areas for the flap, reducing the likelihood of flap failure.

## Conclusion

The PIA flap is a safe, predictable, and versatile option to cover defects in the hand and distal forearm, especially the dorsum, first commissure, and thumb. The morbidity is lower compared with that of other local and microsurgical flaps, resulting in shorter operative time and hospital stay, early rehabilitation, good esthetic acceptance by the patients, and good postoperative outcomes.
